# Shadow AI in Consumer Health: The Case for Safe Adoption of National Health AI Assistants

**DOI:** 10.1016/j.mcpdig.2026.100352

**Published:** 2026-03-11

**Authors:** Darren Y. Chua, Bettina M. McMahon, Simon Kos

**Affiliations:** a Data Science Institute, Faculty of Engineering and IT, University of Technology Sydney, Ultimo, NSW, Australia; b Healthdirect Australia, Sydney, NSW, Australia; c Heidi Health, Melbourne, VIC, Australia

As recently discussed by Singh et al^1^ in Mayo Clinic Proceedings: Digital Health, the Food and Drug Administration (FDA’s) regulatory framework for clinical software is evolving to address the complexities of artificial intelligence (AI). While the FDA is adopting a total product lifecycle approach to regulate AI as software as a medical device (SaMD), generative AI systems challenge these frameworks. Unlike traditional SaMD, which operates within predictable probabilistic bounds, generative AI systems are stochastic, have the capacity to learn, and the way they reach their recommendations is often opaque.^2^

The health care AI environment continues to evolve rapidly; in January 2026, OpenAI and Anthropic launched purpose-built health AI assistants: ChatGPT Health and Claude for Healthcare, respectively.^3^^,^^4^ Although consumers have long relied on ‘Dr Google’, AI assistants offer a more sophisticated alternative through conversational, seemingly personalized advice in a form consumers can understand. These tools could improve access to appropriate services by providing better initial guidance. Yet, notable risks persist. The recent removal of certain health-related summaries from Google AI Overviews following safety concerns highlights the risks of unmonitored consumer-facing AI.^5^ However, as regulators focus on high-stakes clinical governance frameworks, a largely invisible and ungoverned risk is rapidly escalating: consumer usage of shadow AI.

Since the release of ChatGPT in November 2022, consumer adoption of AI in their everyday lives has increased rapidly, with OpenAI reporting 800 million monthly active users on ChatGPT and over 230 million people using ChatGPT specifically for health and wellness questions each week.^3^^,^^6^ Consumers increasingly report using large language models (LLMs) for health-related queries—interpreting symptoms, planning treatment, adopting preventative behaviors, and interpreting test results—with uptake ranging from 9.9% in Australia to 32.6% in the United States.^7^^,^^8^ In this article we refer to this consumer use of LLMs as use of AI assistants.

From a public health perspective, consumer usage of AI assistants for health advice largely occurs outside formal clinical pathways and governance mechanisms; that is, it is unsanctioned by health systems. This pattern parallels shadow IT in organizations, where users adopt unapproved technologies to bridge gaps between their needs and the capabilities of sanctioned systems. Shadow IT is perceived to enable innovation, faster adoption of new technologies, and overcoming limitations of existing systems^9^; however, it exposes organizations to cybersecurity, interoperability, and other risks until it is formally acknowledged and governed.

## Shadow AI

In a similar vein, we use the term shadow AI to describe the adoption of AI assistants operating outside the formal governance and oversight of public health authorities. Although both clinicians and consumers may engage in this practice, this article focuses on the drivers and challenges of shadow AI among consumers.

So why are consumers turning to these tools? Efficacy, convenience, personalization, privacy, perceived empathy, and the ability to bridge access gaps are among the most frequently cited reasons for using AI assistants.^10^, ^11^, ^12^ These tools represent a relevant evolution over traditional noncurated web search results, as consumers value the AI’s ability to synthesize complex information and its perceived empathy. Two studies of Google’s Articulate Medical Intelligence Explorer found that AI assistants not only improved differential diagnoses in complex cases compared with human physicians but were also rated highly by patient-actors on 25 of 26 dimensions, including perceived politeness, ability to put patients at ease, and rapport building.^7^^,^^8^ Adoption for mental health support is also substantial: in a recent study, 48.7% of AI assistant users reported using them for psychological support to address anxiety and depression, and 63.4% reported improved mental health conditions.^11^

### The Liability Gap

The promise of positive impact has led many LLM developers to highlight health care use cases, as exemplified by Microsoft’s MAI-DxO performance on complex cases,^13^ along with OpenAI and Anthropic’s public releases of ChatGPT Health and Claude for Health care.^3^^,^^4^ However, while vendors promote—and consumers adopt—AI assistants, responsibility for harm arising from their use remains largely unaddressed.

Risks include hallucinations, bias, fairness concerns, privacy breaches, automation bias, and over-reliance; in health care, these risks can have serious consequences.^14^^,^^15^ Companies offering AI assistants acknowledge these limitations and explicitly state in their terms of use policies and liability limits that their tools are intended for general information only and do not constitute medical or professional advice (see Table).

**Table:** LLM Provider Terms of Service Disclaimers for General-Purpose AI Assistants

	Consumer usage for medical/health care advice	User input of sensitive/health data	Partner developer responsibilities regarding medical/health care use
OpenAI (ChatGPT)	Users agree that: They should not rely on output from our services as a sole source of truth or factual information, or as a substitute for professional advice. They must evaluate output for accuracy and appropriateness for their use case, including using human review as appropriate. Any use of outputs from our service is at the user’s sole risk. OpenAI is indemnified for damages up to an aggregate liability of the greater of 12 months fees or $100.	Users agree that OpenAI may use content to provide, maintain, develop, and improve our services in essence to train their models. Users have the right to opt out typically by turning off their chat history to ensure privacy.	Prohibited from collecting personal data outside applicable laws and collecting sensitive identifiers. Prohibited from providing tailored legal, medical/health, or financial advice and making automated decisions that affect an individual’s well-being.
Google (Gemini)	Users accept that they should not rely on the services for medical, legal, financial or other professional advice. Any content regarding those topics is provided for informational purposes only and is not a substitute for advice from a qualified professional. Google’s liability is limited to the greater of (1) US $500 or (2) 125% of fees paid in the prior 12 months.	Users have no obligation to provide any content to our services and you’re free to choose the content that you want to provide. If you choose to upload or share content, please make sure you have the necessary rights to do so and that the content is lawful.	Prohibited from engaging in unlicensed practice of health professional services. Prohibited from enabling end users to use the generative AI services for clinical purposes, as a substitute for professional medical advice, or in any manner that requires regulatory approval.
Anthropic (Claude)	Users acknowledge and agree that: Outputs may not always be accurate and may contain material inaccuracies even if they appear accurate because of their level of detail or specificity. Actions may not be error free or operate as intended. They should not rely on any outputs or actions without independently confirming their accuracy. Anthropic’s aggregate liability is limited to the greater of fees paid over the preceding 6 months or $100.	Users are responsible for all inputs that they submit to the service and resultant actions. Users provide a warranty and indemnity that the use of services and actions is at their risk.	Developers are required to implement safety measures for high-stakes use cases including healthcare, medical diagnosis and mental health. Safety measures include: Human-in-the-loop involving a qualified professional to review content and accuracy. Disclosure of the use of AI in producing advice or decisions. Disclosure for consumer-facing chatbots that users are interacting with AI rather than a human.
X.AI (Grok)	Users understand and agree: That output may not always be accurate and does not constitute professional advice. Users should conduct thorough research and should not rely on output as the truth. They are responsible for evaluating output for accuracy and appropriateness for use, including using human review and supervision, before using or sharing output. That AI is rapidly evolving and is probabilistic in nature and can result in output, including hallucinations. X.AI is indemnified for damages relating to the use of the service and for costs exceeding the greater of the amount paid to X.AI or $100.	Users may provide input to the service and are responsible for their content. Users can elect whether their content is used for model training.	Prohibited from enabling users to submit sensitive personal data under privacy laws or protected health information under HIPAA privacy rules. Required to evaluate the accuracy of any output as appropriate for the customer’s use case, including by using human review of the output.
DeepSeek	Users agree that: outputs provided by the service are generated by an artificial intelligence model and may contain errors or omissions and are for reference only. Users should not treat the Outputs as professional advice. Specifically, when using this service to consult on medical, legal, financial, or other professional issues, they should be aware that the service does not constitute any advice or commitment and does not represent the opinions of any professional field. They should consult professionals and make decisions under their guidance.	Users are responsible for all inputs they submit to the service and for corresponding outputs. Users also indemnify DeepSeek against liabilities and damages arising from themselves breaching the terms of use.	Responsible for obtaining user consents and ensuring legal basis for the processing of information in alignment with the terms of use. Warrant that they have all rights, licenses, and permissions that are necessary for DeepSeek to process the Inputs under their stated terms.

Abbreviations: AI, artificial intelligence; LLM, Language large model.

A liability gap has emerged whereby general-purpose AI assistants are promoted and adopted by consumers at scale for health care purposes, yet the risks of errors are borne almost entirely by consumers. This risk is compounded by the absence of systematic, evidence-based research on consumer use of these now ubiquitous tools, given the speed of adoption. The true scale of the problem remains unknown.

### Regulation and the Intended Use Dilemma

The FDA and international agencies such as the UK’s Medicines and Health care products Regulatory Agency, and Australia’s Therapeutic Goods Administration each regulate AI tools as SaMD—similar to drugs and devices—rather than taking a system approach to the safety and efficacy of such tools.^2^ However, this oversight hinges on specific definitions of intended use. As Singh et al^1^ note, current FDA guidelines explicitly exclude tools intended for administrative tasks and general health and wellness from SaMD oversight.

This distinction creates a paradox: companies offering general-purpose AI assistants effectively position them within these regulatory carve-outs by explicitly disclaiming medical advice within their terms of service. Yet, despite these restrictions, consumers are using these tools for diagnostic and treatment purposes that would normally demand rigorous SaMD review with health system oversight and governance.

Medical device certification typically requires a ‘locked’ scope, assuming a product is implementation ready.^2^ By contrast, AI assistants are developed iteratively through test-and-learn cycles, co-design and experimentation informed by data from the experience.^16^ These agile methods are prevalent in technology design and offer a way to manage dynamic uncertainty; appropriate for AI assistants that fall outside traditional SaMD definitions and exhibit an innately plastic nature—evolving based on user inputs and changing contexts.^17^

Currently, there is no graduated ‘on-ramp’ for AI assistants yet to reach SaMD status. Iterative development will likely push these tools into SaMD territory as consumers demand features for treatment advice and monitoring and as governments deploy AI assistants to meet demand for health advice amid workforce constraints.

### Government Response

Just as corporate IT departments lost the battle to prohibit shadow IT and ended up developing policies to guide its use, governments are beginning to lean into consumer demand for AI assistants. Two major economies have announced plans to release government-backed tools. In July and August 2025, the US Centers for Medicare & Medicaid Services (CMS) and the English National Health Service (NHS) announced plans to release AI assistants to their citizens. CMS intends these systems to help patients check symptoms, navigate care options, and schedule appointments.^18^ The NHS application expands scope to allow booking and rescheduling appointments, self-referral, integration with wearable devices, while using AI to provide instant advice for non-urgent care.^19^

This is a logical extension of the trend towards virtual front doors that triage and connect consumers with appropriate care for their conditions, reduce low-value interactions or overservicing, and improve consumer convenience and system productivity by automating administrative tasks like bookings.^20^ The announcements suggest a consumer experience incorporating features of agentic AI to personalize interactions and add a conversational layer. Although scope and implementation details remain unclear, governments need to consider the graduated approach to safety as they move from administrative tasks and triage towards care management and potentially autonomous referral into digital care pathways.

The emergence of private health AI assistants also raises questions about whether governments should compete with industry or leverage it. National systems could potentially enable commercial platforms to access health records held in public systems, subject to rigorous safety, security, and governance standards. Such approaches warrant careful further exploration.

### Proportionate Steps up an SaMD On-Ramp

With consumer AI already widespread and governments embracing this trend in their national plans, a more adaptive governance model is needed to complement SaMD regulation—one that mitigates risks from unregulated AI assistants while enabling health care agencies to test and deploy safer services. One progressive example is the German Federal Institute for Drugs and Medical Devices, which includes a fast-track option for provisional certification of digital health applications under the DiGA framework, in which the regulator has three months to review product qualities and healthcare benefits, and if successfully validated, the application can proceed to a 12 month trial.^21^

We propose a phased and adaptive approach involving targeted risk-based pilots, AI sandboxes and evidence-based evaluations. A recent example enacted by the UK Medicines and Health care products Regulatory Agency is the HealthAI Global Regulatory Network which includes AI Airlock, a regulatory sandbox for AI medical device testing.^22^ Agile approaches could recruit and closely monitor consumers starting with lower-risk administrative tasks to higher risk, higher benefit features.

These pilots would facilitate the development of healthcare-specific evaluations to measure clinical accuracy, safety, efficiency, and consumer experience (eg, anxiety and diagnostic understanding). Industry-developed quality benchmarks such as OpenAI’s HealthBench and Stanford’s MedAgentBench provide valuable foundations for measuring safety and accuracy of health AI assistants. They are a practical first step from technical capability up an on-ramp toward regulated deployment. A second step could involve lite certification pathways for AI assistants that extend beyond benchmark validation to include additional clinical features or decision support capabilities. Iterative refinements would be informed by consumer feedback, safety outcomes, and behavioral insights.

The proposed approach provides a graduated regulatory pathway for both public health AI assistants (eg, CMS, NHS) and private tools (eg, ChatGPT Health or Claude for Healthcare) that seek integration with formal health care systems. The SaMD on-ramp approach directly addresses the intended use dilemma and the liability gap by providing the exclusive gateway to reimbursement, clinical data integration, and funded health services. Private developers would be incentivized to submit their tools to this oversight to unlock commercial value through integration with patient records and public health infrastructure.

To succeed, this framework requires legislative reforms that empower regulators to manage total system safety rather than isolated devices. Participation in the on-ramp would require developers to establish technical guardrails such as mandatory escalations to human clinicians for high-stakes queries, along with commitment to a shared liability model. This effectively shifts the burden of risk away from the individual consumer and toward a shared governance model between developers, providers, and health systems (Table).^3^^,^^4^^,^^23^, ^24^, ^25^, ^26^, ^27^, ^28^, ^29^, ^30^, ^31^, ^32^

It seems likely that AI health assistants will disrupt existing human-in-the-loop models as consumers increasingly use them as their first port-of-call for health queries, and health systems seek to shift low acuity care to digital channels, deploying the resource-constrained human workforce further toward operating at the top of scope. A mixed-model care team could emerge that involves the consumer with an AI assistant accessing their health data and providing evidence-based self-care advice and an initial entry point into the health system. For consumers with higher health needs such as those living with chronic disease, the consumer would be supported by a care team, with the AI assistant improving coordination and continuity of care.

Governance and liability frameworks must adapt, incorporating new human-in-the-loop mechanisms and shared responsibility among healthcare providers, health systems providing AI assistants, AI developers, and consumers themselves.

## Conclusion

Although the SaMD on-ramp offers a pathway for safe adoption, important implementation challenges exist. Given current commercial race dynamics, major AI developers (eg, OpenAI, Anthropic, and Google, X) are highly incentivized to maximise deployment speed over the governance required by health systems. Developers may hesitate to engage with regulatory sandboxes if they perceive them as barriers to speed. Furthermore, shadow AI is likely to persist due to its inherent convenience; regulation alone cannot stop adoption. However, by positioning nationally sanctioned health AI assistants as the exclusive, trusted gateway to reimbursement, clinical data integration and specialist escalation, the proposed approach offers a superior value proposition that incentivizes a shift away from unregulated tools toward sanctioned, evidence-based care.

New mindsets will be needed to avoid perfection getting in the way of good. Regulators should shift from a device-centric mindset toward iterative and graduated risk-based implementation to reduce system-wide consumer harm across the SaMD on-ramp.

Health care systems should accelerate deployment of nationally guided, trustworthy AI assistants, beginning with administrative functions and health alerts. Crucially, regulators and healthcare systems will need to collaborate on optimized reimbursement mechanisms for sanctioned access to integrated models of care. This approach will, in turn, incentivize AI developers to engage in transparent, collaborative partnerships with health system leaders.

## Potential Competing Interests

Ms McMahon is the Chief Executive Officer of Healthdirect Australia and is a Director on the Board of SNOMED International. Dr Kos is the Global Chief Medical Officer of Heidi Health, and contributed to early drafts while employed by Microsoft before he joined Heidi Health. Mr Chua is a PhD candidate at the University of Technology Sydney, undertaking unfunded research in collaboration with Healthdirect Australia. Mr Chua and Dr Kos are co-founders of Lumyra, an AI governance advisory firm. All authors have completed the ICMJE uniform disclosure form.

## Ethics Statement

Ethics approval was not required for this commentary as it relies on publicly available information and does not involve human subjects or patient data.

## Declaration of Generative AI and AI-Assisted Technologies in the Writing Process

During the preparation of this work the authors used Microsoft Copilot, Gemini and Claude in order to improve readability and formatting. After using these tools, the authors reviewed and edited the content as needed and takes full responsibility for the content of the publication.
